# Educational strategies related to information science and technology for medical students: General medicine physicians' perspective

**DOI:** 10.1002/jgf2.656

**Published:** 2023-11-06

**Authors:** Kiyoshi Shikino, Masaki Tago, Takashi Watari, Yosuke Sasaki, Hiromizu Takahashi, Taro Shimizu

**Affiliations:** ^1^ Department of General Medicine Chiba University Hospital Chiba Japan; ^2^ Department of General Medicine Saga University Hospital Saga Japan; ^3^ General Medicine Center Shimane University Hospital Izumo Shimane Japan; ^4^ Department of General Medicine and Emergency Care Toho University School of Medicine Tokyo Japan; ^5^ Department of General Medicine, Faculty of Medicine Juntendo University Tokyo Japan; ^6^ Department of Diagnostic and Generalist Medicine Dokkyo Medical University Tochigi Japan

## Abstract

The Model Core Curriculum for Medical Education in Japan was revised in 2022. It aimed to reflect changes in the nature of medical care, including the advancement of medical technology through the use of information science and technology and artificial intelligence in the Society 5.0 era. We summarize recommendations for good practice regarding learning strategies from the perspective of general medicine.

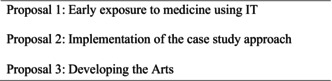

To the Editor,

The Model Core Curriculum for Medical Education in Japan was revised in 2022.^1^ It aimed to reflect changes in the nature of medical care, including the advancement of medical technology through the use of information science and technology (IT) and artificial intelligence (AI) in the Society 5.0 era.

“IT” has been incorporated into this updated version.^1^ Educational goals compatible with general medicine have been integrated, like the adaptation of information science and technology, including hospital management and work efficiency. It is also included in the competencies at the society of hospital medicine in the United States.^2^ However, specific learning strategies must be implemented. We summarize recommendations for good practice regarding learning strategies from the perspective of general medicine (Table 1).

**TABLE 1:** Three proposal educational strategies related to information science and technology for medical students.

Proposal 1: Early exposure to medicine using IT
Proposal 2: Implementation of the case study approach
Proposal 3: Developing the Arts

Proposal 1: *Early exposure to medicine using IT*. Learners must understand the intention behind studying IT and improve their readiness. One effective means is early exposure, which gives students a concrete image and improves their learning motivation by providing them with various experiences from an early stage.^3^ For example, students can join clinical settings where IT is being used, such as vast amounts of medical information being directly connected to medical care and quality control. They can also experience telemedicine and EBM using digital applications through IT.

Proposal 2: *Implementation of the case study approach*, which is a problem‐solving research method.^4^ Specific cases are closely analyzed to derive the general rules and principles necessary for problem‐solving. This can foster real‐world problem‐solving skills that cannot be obtained through studying theory alone. Specific examples include proficiency in the use of research engines, exposure to AI literature search services, and information literacy. The use of IT in the case study contributes to efficient and effective medical treatment and improvements in medical care. Cultivating an ethical viewpoint to deal with IT is crucial because they are imperative in dealing with patient information, even in the context of digital medicine.

Proposal 3: *Developing the Arts*. In medicine, there is a focus on the use of generative AI.^5^ In this context, it is necessary to pursue and develop the value that only humans can bring to the table, such as activities in the conceptual work process, ability to learn and adapt to the output of generative AI, and other strengths. If we call AI and other technologies Science, then Art is the complementary concept within human expertise. Arts and Science have many ways in which they intersect and influence each other. For nurturing Arts, it is important to focus on process rather than solely on the outcome. This means that, in view of Arts and Science collaboration, studying AI process (Science) will enhance the quality of human expertise (Arts). AI is widely used by generalists, enabling the application of this augmentation theory. As general medicine significantly contributes to medical education, this aspect is necessary in curriculum development. Specific examples include patient interaction with empathy, ethical decision‐making, and continuous learning and adaptation.

General medicine has become more central to the educational practices of the curriculum in Japan. The authors believe that the future of medical education will improve significantly because of its collaboration with general medicine.

## CONFLICT OF INTEREST STATEMENT

The authors have stated explicitly that there are no conflicts of interest in connection with this article.

## References

[1] Medical Education Model Core Curriculum Coordination Committee, Medical Education Model Core Curriculum Expert Research Committee . Model core curriculum for medical education. AY 2022 revision. 2023. Available from: https://www.mext.go.jp/b_menu/shingi/chousa/koutou/116/toushin/mext_01280.html. Accessed 3rd Nov 2023.

[2] Nichani S , Fitterman N , Lukela M , Crocker J , Society of Hospital Medicine . The core competencies in hospital medicine 2017 revision. Section 3: healthcare systems. J Hosp Med. 2017;12(4 Suppl 1):S55–S82. 28411301 10.12788/jhm.2729

[3] Murphy F . Motivation in nurse education practice: a case study approach. Br J Nurs. 2006;15(20):1132–1135. 17170664 10.12968/bjon.2006.15.20.22300

[4] Lawson McLean A , Saunders C , Velu PP , Iredale J , Hor K , Russell CD . Twelve tips for teachers to encourage student engagement in academic medicine. Med Teach. 2013;35(7):549–554. 23496123 10.3109/0142159X.2013.775412

[5] Tan TF , Thirunavukarasu AJ , Jin L , Lim J , Poh S , Teo ZL , et al. Artificial intelligence and digital health in global eye health: opportunities and challenges. Lancet Glob Health. 2023;11(9):e1432–e1443. 37591589 10.1016/S2214-109X(23)00323-6

